# Movement behaviour education for parents in prenatal, postnatal, and pediatric care in Canada: A needs assessment

**DOI:** 10.1186/s12887-024-04630-4

**Published:** 2024-03-08

**Authors:** Brianne A. Bruijns, Matthew Bourke, Aidan Loh, Patricia Tucker

**Affiliations:** 1 School of Occupational Therapy, Faculty of Health Sciences, Western University, 1201 Western Road, Elborn College, Room 2547, London, ON N6G 1H1 Canada; 2 Children’s Health Research Institute, London, ON Canada

**Keywords:** Movement behaviours, Prenatal care, Postnatal care, Pediatric care, Physical activity, Sedentary behaviour, Sleep, Early childhood

## Abstract

**Background:**

Parents/guardians can greatly influence their child’s movement behaviours (i.e., physical activity, sedentary behaviour, and sleep). Yet, they have reported to lack sufficient background knowledge to foster healthy movement habits, and little is known about specific educational gaps. The aim of this study was to explore the educational background and needs regarding promoting healthy movement behaviours in early childhood among parents/guardians living in Canada.

**Methods:**

A cross-sectional study was conducted with parents/guardians living in Canada who had at least one child under the age of 5 years. Participants completed an online survey capturing their demographics, information they received about movement behaviours in early childhood during their prenatal/postnatal care or child’s pediatrician appointments, where they sourced information about these topics, content areas they would like more information on, and preferred format for delivery. Descriptive statistics and frequencies were calculated for all outcome variables and logistic regression was used to explore if sociodemographic variables were associated with receiving movement behaviour-related education across care types.

**Results:**

Among the 576 parents/guardians who completed the survey, many reported no mention of any movement behaviour in their prenatal (49.4%), postnatal (29.6%), and pediatric care (37.2%). Physical activity was the most cited movement behaviour across care types, with 42.4%, 57.9%, and 54.8% of participants indicating this was discussed in their prenatal, postnatal, and pediatric care, respectively. Only 41.7% of parents/guardians reported asking their child’s pediatrician about movement behaviours, while most relied on social media (70.9%), internet websites/news articles (68.7%), and family/friends (67.6%). The most sought-after movement behaviour topics included incorporating movement into traditionally sedentary activities (68.8%) and activity ideas to break up sitting time (65.0%), and participants expressed preference to receive more information via social media (63.2%), an online resource package (47.8%), or email (46.6%).

**Conclusions:**

Given the noted inconsistencies in education relating to movement behaviours in maternal and pediatric care, this study highlights the opportunity for greater integration of this type of education across care types. Ensuring all parents/guardians receive evidence-based and consistent guidance on their child’s movement behaviours will help ensure young children receive the best start to a healthy active life.

**Supplementary Information:**

The online version contains supplementary material available at 10.1186/s12887-024-04630-4.

## Introduction

In 2017, the Canadian Society for Exercise Physiology (CSEP) introduced a resource called the *Canadian 24-Hour Movement Guidelines for the Early Years (0–4 years)* [1]. These guidelines were based on the best available evidence of the multitude of health and developmental benefits of engaging in healthy movement behaviours (i.e., engaging in physical activity, limiting sedentary behaviour, and getting enough sleep) [1, 2]. For infants (< 1 year), guidelines suggest being physically active through interactive floor-based play, including 30 minutes of tummy time per day, and that they should not be sedentary or restrained for more than 1 hour at a time, nor should they engage in any screen time. For sleep, infants under 4 months should sleep for 14 to 17 hours, and those aged 4 to 11 months should sleep for 12 to 16 hours per day, including naps [1]. For toddlers (1–2 years old) and preschoolers (3–4 years old), the guidelines suggest a minimum of 180 minutes of total physical activity each day, and for preschoolers, 60 of those minutes should be in energetic play (i.e., moderate-to-vigorous physical activity [MVPA]). Further, toddlers and preschoolers should not be sedentary or restrained for more than 1 hour at a time and only children 2 years of age and older should engage in any screen time (and this should be limited to no more than 1 hour per day) [1]. Finally, toddlers should get 11 to 14 hours of quality sleep daily, while preschoolers should get 10 to 13 hours per day, with consistent bed and wake-up times. These guidelines have since been adopted by the World Health Organization [2], and by many other countries, [3–5] to increase the proportion of young children worldwide who exhibit healthy 24-hour movement behaviour profiles.

A recent review and meta-analysis by Tapia-Serrano and colleagues [6] aimed to evaluate adherence to the overall 24-hour movement guidelines for preschoolers (3–4 years), children (5–11 years), and adolescents (12–18 years) across the world. A total of 63 studies comprising 387,437 children and youth were included in the review, and findings showed that the proportion of preschoolers achieving all movement behaviour guidelines was only 11.3%. Similar estimates were found among a sample of Canadian toddlers (*n* = 151), with only 11.9% meeting the overall 24-Hour Movement Guidelines; [7] however, this finding was largely driven by screen time, with only 15.2% of toddlers meeting this recommendation. Another Canadian research study [8] used a parent-reported questionnaire to assess infants’ adherence to the 24-hour movement guidelines and found that only 2% of the sample (*n* = 250) reported that their infant met all three behaviour recommendations. These findings were similar to a study among parent-infant dyads (*n* = 455) in Australia [9], which reported that only 3.5% of infants met the combined guidelines. Given the consistently low reported adherence to the 24-hour guidelines, more comprehensive movement behaviour interventions may be needed to support engagement in healthy 24-hour movement behaviours among children in the first 2000 days of life.

Young children spend a significant amount of time in the care of their parents or legal guardians [10], which suggests initiatives targeting these individuals are essential in the promotion of healthy movement behaviours. Parents play an important role in the development of children’s movement behaviours and can influence children’s active play through encouragement, support, and modelling [11]. Given the important role that parents can play, an expert panel developed a consensus statement highlighting the role of the family in supporting children and youth to achieve healthy physical activity, sedentary, and sleep behaviours [12]. This paper included a series of key evidence statements that outlined the important role of parental modelling, emotional support, knowledge, and expectations on healthy physical activity and sedentary behaviours [12], necessitating the value of ensuring parents and guardians are well-informed of movement behaviour guidelines and best practices in the early years.

Although there is strong evidence that outlines the important role parents play in engaging young children in healthy movement behaviours [11, 12], research suggests there is a gap in knowledge among parents and guardians in this area [9, 13]. For example, parents and guardians may not even be aware of the 24-hour movement behaviour guidelines for young children [14], they may lack knowledge regarding strategies to assist themselves in facilitating infant adherence to the guidelines [9], and they may not be aware of the importance of meeting the 24-hour movement guidelines for young children’s health [15]. Therefore, it is clear that greater efforts are needed to raise parents’ and guardians’ awareness of the importance of healthy physical activity, sedentary behaviour, and sleep in early childhood and the role they play in supporting their children to meet recommendations [14]. What is presently missing from the literature is a fulsome picture of parents’ and guardians’ educational background and needs across all movement behaviours and age ranges (i.e., infants, toddlers, preschoolers) in early childhood, including where this information is coming from (e.g., prenatal/postnatal care, child’s pediatrician/doctor) [16] and how much detail is provided. This gap needs to be addressed to best support them in the promotion of healthy movement behaviours for children [17].

This research seeks to fill the above-mentioned gap in understanding the educational background and needs of parents and guardians in effectively promoting healthy movement behaviours in young children. Therefore, the aim of the Movement Education for parents of YOUng children (ME & YOU) needs assessment study was to explore parents’ and guardians’ educational background (i.e., information received in their prenatal, postnatal, and child’s pediatric care) and needs (i.e., topics they would like to know more about) regarding promoting healthy movement behaviours in early childhood among those living in Canada. A secondary objective of the study was to explore sociodemographic associations with parents’ and guardians’ educational background in movement behaviours. To our knowledge, this is the first study to provide a more fulsome and holistic understanding of the reported gap in movement behaviour education among parents and guardians of young children in Canada and will inform educational resource development and training for this group, as well as the practice of primary care practitioners working with new parents and young children.

## Methods

A cross-sectional mixed methods study design was employed for the ME & YOU needs assessment, which was approved by the Non-Medical Research Ethics Board at Western University (REB# 122865).

### Study procedures and participant recruitment

Parents/guardians living in Canada, with at least one child under the age of 5 years and who could read and write in English or French, were recruited via social media advertisements (e.g., Twitter, Instagram) and recruitment flyers distributed via email to members of various parenting/family organizations from May to August 2023. Implied consent was given by commencing the survey.

#### Online survey

An online survey was developed and administered via Qualtrics in both English and French for the purposes of this study. Four items were used to ask if parents/guardians received information about physical activity, sedentary behaviour and/or sleep in early childhood during their prenatal/postnatal care or child’s doctor/pediatrician appointments, and if they sourced their own information about these topics (e.g., online, via apps, asking family or friends). Five items were used to gather participants’ perspectives about physical activity, sedentary behaviour, sleep, and outdoor play content areas they would like to learn more about, and the format they would like to use to access this information. Additionally, 15 items were used to gather participants’ demographics, including their: age; gender; racial background; province/territory of residence; family situation (i.e., single-parent, double-parent, guardian-led, other); number and ages of their child(ren); the care arrangement for their child(ren); personal levels of physical activity and recreational screen time; highest level of education; employment status; annual household income; and, housing type.

### Data analysis

Descriptive statistics were calculated in SPSS (version 29) to analyze quantitative data from the online survey. Means (*M*) and standard deviations (*SD*) were used to calculate participant age, while frequencies were used to analyze all remaining quantitative data. Text responses to survey questions were collapsed in Microsoft Excel Workbook and analyzed in QSR NVivo, and frequencies of movement behaviour-related topics covered in participants’ prenatal, postnatal, and pediatric care were calculated. Logistic regression analysis was conducted to explore if any sociodemographic variables were associated with receiving physical activity, sedentary behaviour, sleep, or no movement related education during prenatal, postnatal, and pediatric care. Overall differences based on sociodemographic variables was estimated using Wald tests, and differences between individual demographic groups were examined using odds ratios (OR) and their 95% confidence intervals (CI) between individual groups.

## Results

### Participant demographics

A total of 576 Canadian parents/guardians of children under 5 years of age were recruited for this study; 529 participants (91.8%) completed the English-language survey and 47 participants (8.2%) completed the French-language survey. Parents/guardians were, on average, 34.4 years old (*SD* = 3.8 years), and the majority were female (94.2%), Caucasian (76.7%), from Ontario (33.8%), British Columbia (16.1%), or Québec (12.1%), and from double-parent households (95.4%). Approximately half (53.6%) of parents/guardians had one child, 34.1% had two children, and 12.3% had 3 or more children. Further, 39.0% of parents/guardians reported having an infant, 52.4% having a toddler, and 47.0% having a preschooler. Less than one-quarter of parents/guardians (22.7%) reported meeting the adult physical activity guideline, while the majority (77.3%) reported to meet the recreational screen time guideline in the *Canadian 24-Hour Movement Guidelines for Adults (18–65 years)*. See Table 1 for complete participant demographics.

**Table 1:** Participant Demographic Information

Variable	M	SD	Variable	N	%
Age	34.4	3.8			
	**N**	**%**	**Number of Children Caring for**		
**Gender**			1	270	53.6
Female	489	94.2	2	172	34.1
Male	27	5.2	3	50	9.9
Non-Binary	3	0.6	4 or more	12	2.4
**Ethnicity**			**Age Category of Child(ren) Under 5 Years**		
Arab	1	0.2	Infant (< 1 year)	187	39.0
Black	10	1.9	Toddler (1 to 2 years)	251	52.4
White	399	76.7	Preschooler (3 to 4 years)	222	47.0
Indigenous Peoples of Canada	17	3.3	**Childcare Arrangement**		
First Nations	10	58.8	Parental Care	167	32.5
Métis	7	41.2	Centre-Based Childcare or Preschool	165	32.1
Inuit	0	0.0	Home-Based Childcare	67	13.0
Latin, Central, or South American	17	3.3	Full-Day Kindergarten	14	2.7
East Asian	34	6.5	Mixed Care Arrangement	101	19.6
South Asian	17	3.3	**Highest Level of Education**		
Southeast Asian	7	1.3	High School	14	2.7
West Asian	2	0.4	College	60	11.7
Mixed Racial Background	12	2.1	University	237	46.2
Prefer not to answer	4	0.8	Graduate School	202	39.4
**Province/Territory**			**Employment Status**		
British Columbia	73	16.1	Full-Time	210	41.0
Alberta	51	11.3	Part-Time	53	10.4
Saskatchewan	35	7.7	Occasional/Support	13	2.5
Manitoba	35	7.7	On Parental Leave from Full-Time Employment	173	33.8
Ontario	153	33.8	Unemployed	29	5.7
Québec	55	12.1	Other	30	5.9
New Brunswick	10	2.2	Prefer not to answer	3	0.6
Nova Scotia	24	5.3	**Annual Household Income**		
Prince Edward Island	7	1.5	Less than $60,000	46	9.0
Newfoundland and Labrador	5	1.1	$60,000 to $120,000	159	31.0
Nunavut	0	0.0	More than $120,000	270	52.7
Northwest Territories	3	0.7	**Housing Type**		
Yukon	2	0.4	Apartment	47	9.2
**Family Situation**			Condominium	36	7.1
Single Parent	23	4.4	Townhouse	68	13.3
Double Parent	495	95.4	Semi-Detached House	44	8.6
Other	1	0.2	Detached House	315	61.8
**Meeting the Adult Physical Activity Guideline** ^**a**^			**Meeting the Adult Screen Time Guideline** ^**a**^		
Yes	116	22.7	Yes	344	67.2
No	396	77.3	No	168	32.8

Note. ^a^ 150 min/week of moderate-to-vigorous physical activity and < 3 h/day of recreational screen time as per the *Canadian 24-Hour Movement Guidelines for Adults* (CSEP, 2020)

### Educational background

#### Prenatal care

Nearly half (49.4%) of parents/guardians reported no mention of early childhood physical activity, sedentary behaviour, nor sleep in their prenatal care (Table 2). The most common movement behaviour discussed in participants’ prenatal care was physical activity (42.4%), followed by sleep (33.0%), and sedentary behaviour (28.3%). Less than one-quarter of parents/guardians (22.6%) reported that their prenatal care provider discussed all three movement behaviours. Of those who reported receiving information on physical activity in early childhood (*n* = 229), 80.8% provided a text response that this included information about infant tummy time. However, some parents/guardians were provided with more information than others. For example, one participant noted receiving education that: “tummy time is important for development of [the] neck and back; start with short durations of 1–2 minutes and increase to 30 minutes a day…”. Another participant mentioned they: “…found the information really lacking and sought out more information on what to do with [their] child through different ages and stages.”

**Table 2:** Information Parents Received in Pre-/Post natal Care and Child’s Pediatrician Appointments About their Child’s Physical Activity, Sedentary Behaviour, and Sleep

**Prenatal Care (N = 540)**	**N**	**%**
Only PA	47	8.7
Only SB	3	9.3
Only Sleep	18	12.6
PA and SB Only	25	17.2
PA and Sleep Only	35	23.7
SB and Sleep Only	5	24.6
PA, SB, and Sleep	122	22.6
Any PA	229	42.4
Any SB	153	28.3
Any Sleep	178	33.0
No mention of any behaviour in care	267	49.4
Did not receive prenatal care	18	3.3
**Postnatal Care (N = 537)**	**N**	**%**
Only PA	76	14.2
Only SB	9	1.7
Only Sleep	27	5.0
PA and SB Only	26	4.8
PA and Sleep Only	68	12.7
SB and Sleep Only	1	0.2
PA, SB, and Sleep	142	26.4
Any PA	311	57.9
Any SB	177	33.0
Any Sleep	234	43.6
No mention of any behaviour in care	159	29.6
Did not receive postnatal care	29	5.4
**Pediatric Care (N = 545)**	**N**	**%**
Only PA	107	19.6
Only SB	9	1.7
Only Sleep	23	4.2
PA and SB Only	28	5.1
PA and Sleep Only	63	11.6
SB and Sleep Only	3	0.6
PA, SB, and Sleep	103	18.9
Any PA	299	54.8
Any SB	141	25.9
Any Sleep	190	34.9
No mention of any behaviour in care	203	37.2
Did not receive pediatric care	6	1.1

Note. PA = physical activity; SB = sedentary behaviour

Less than half (43.1%) of participants receiving information on sedentary behaviour in early childhood (*n* = 153) noted that this included limiting time sedentary or restrained, and few (17.6%) mentioned receiving any information about limiting screen time (Table 2). Even those receiving this education highlighted that this information was often limited; one participant noted that “in [their] prenatal course [they] were told too much container time can be harmful, but very few details were provided.” Moreover, conflicting screen time recommendations were shared, with some parents/guardians reporting receiving guidance of “no screen time for children under the age of 3”, while others indicating their care provider mentioned “no screen time under 2 years” or “…under 18 months”.

Of the parents/guardians who reported receiving information about sleep in early childhood in their prenatal care (*n* = 178), less than half (43.3%) elaborated that this included information about 24-hour sleep recommendations, and the level of detail varied (Table 2). For example, one participant shared that they received information that “daily sleep total matters more than length of nap, starting around 12–18 hours a day, generally broke up over 24hr period”, while another shared that they were “taught that sleep in newborns is unpredictable [but] did not get a lot of detail.” Some participants receiving sleep-related guidance (22.5%) also discussed learning about safe sleep recommendations, such as “back to sleep” and “no extra things in [the] crib.”

Only participants’ highest level of education was significantly associated with the proportion of participants receiving physical activity-related education (χ2 = 11.74, *p* = .008), or no movement behaviour-related education (χ2 = 9.249, *p* = .026) in their prenatal care (Table 3). Interestingly, participants who completed college programs demonstrated greater odds of receiving physical activity-related education compared to participants who attended graduate school (OR = 2.824, 95%CI = 1.525, 5.229, *p* < .001) or had an undergraduate degree (OR = 2.654, 95%CI = 1.449, 4.864, *p* = .002). Additionally, those with a college degree were at significantly lower odds of not receiving any movement behaviour-related education compared to participants who attended graduate school (OR = 0.389, CI = 0.209, 0.723, *p* = .003), and parents with an undergraduate university degree (OR = 0.441, 95%CI = 0.240, 0.812, *p* = .009) in their prenatal care (see Appendix A for all ORs).

**Table 3:** Influence of Sociodemographic Characteristics on Participants’ Movement Behaviour-Related Education Received in their Prenatal, Postnatal, and Pediatric Care

	**Prenatal Care**			
	**Physical Activity**	**Sedentary Behaviour**	**Sleep**	**None**
Ethnicity	χ^2^(1) = 1.265, *p* = .264	χ^2^(1) = 0.915, *p* = .339	χ^2^(1) = 1.607, *p* = .205	χ^2^(1) = 2.214, *p* = .137
Income	χ^2^(3) = 3.584, *p* = .310	χ^2^(3) = 3.533, *p* = .316	χ^2^(3) = 0.716, *p* = .869	χ^2^(3) = 3.428, *p* = .330
Education	**χ**^**2**^**(3) = 11.738,** ***p*= .008**	χ^2^(3) = 5.338, *p* = .149	χ^2^(3) = 4.290, *p* = .232	**χ**^**2**^**(3) = 9.249,** ***p*** **= .026**
Province	χ^2^(7) = 7.584, *p* = .371	χ^2^(7) = 9.093, *p* = .246	χ^2^(7) = 6.000, *p* = .540	χ^2^(7) = 6.274, *p* = .508
	**Postnatal Care**			
	**Physical Activity**	**Sedentary Behaviour**	**Sleep**	**None**
Ethnicity	χ^2^(1) = 0.057, *p* = .811	χ^2^(1) = 0.705, *p* = .401	χ^2^(1) = 0.123, *p* = .726	χ^2^(1) = 0.005, *p* = .943
Income	χ^2^(3) = 2.389, *p* = .496	χ^2^(3) = 1.047, *p* = .790	χ^2^(3) = 0.492, *p* = .921	χ^2^(3) = 2.343, *p* = .504
Education	χ^2^(3) = 3.908, *p* = .272	χ^2^(3) = 3.011, *p* = .390	χ^2^(3) = 7.417, *p* = .060	χ^2^(3) = 6.293, *p* = .098
Province	χ^2^(7) = 7.618, *p* = .367	χ^2^(7) = 5.890, *p* = .553	χ^2^(7) = 10.063, *p* = .185	χ^2^(7) = 6.521, *p* = .480
	**Pediatric Care**			
	**Physical Activity**	**Sedentary Behaviour**	**Sleep**	**None**
Ethnicity	χ^2^(1) = 0.054, *p* = .816	χ^2^(1) = 0.404, *p* = .525	χ^2^(1) = 5.527, *p* = .019	χ^2^(1) = 1.332, *p* = .194
Income	χ^2^(3) = 2.422, *p* = .490	χ^2^(3) = 3.757, *p* = .289	χ^2^(3) = 6.458, *p* = .091	χ^2^(3) = 3.739, *p* = .291
Education	χ^2^(3) = 1.221, *p* = .748	χ^2^(3) = 4.482, *p* = .214	χ^2^(3) = 3.397, *p* = .334	χ^2^(3) = 3.138, *p* = .371
Province	χ^2^(7) = 12.664, *p* = .073	**χ**^**2**^**(7) = 17.707,** ***p*** **= .013**	χ^2^(7) = 10.326, *p* = .171	**χ**^**2**^**(7) = 19.788,** ***p*** **= .006**

Note. Results presented as: odds ratio (95%CI), *p*-value; significance set at *p* < .05

#### Postnatal care

In participants’ postnatal care, most (57.9%) reported that their provider discussed early childhood physical activity, while less than half reported the mention of sedentary behaviour (33.0%) or sleep (43.6%; Table 2). Just under one-third (29.6%) of parents/guardians reported no mention of any movement behaviour in their postnatal care, while 26.4% reported that all three movement behaviours were discussed. Of parents/guardians who reported receiving physical activity information in their postnatal care (*n* = 311), most (70.1%) elaborated that this included information on tummy time. Other physical activity topics mentioned included: “physical milestones baby should meet at checkups” and “how to encourage reaching for objects.”

Similar to participants’ prenatal care, less than half (42.4%) of those who received information in their postnatal care on sedentary behaviour explained that this captured information on minimizing time spent sedentary or restrained. Further, only some participants (20.3%) noted the mention of screen time; yet, again, there was discrepancy in guidance from health professionals, with some noting they were instructed to provide no screen time under 12 months, while others reported this limitation up to 3 years old. Some participants emphasized the lack of information about sedentary behaviour by their care provider; for example, one participant noted “[they] researched this on [their] own…and took an active role in seeking out information about infant movement affected by too much time in containers…”.

Of the parents/guardians who reported receiving information about sleep in early childhood in their postnatal care (*n* = 234), only 22.6% elaborated that this included information about 24-hour sleep recommendations, while 24.8% discussed the mention of safe sleep practices. Many parents also discussed the mention of wake/feed cycles in the first few months postpartum; for example, one participant noted they “were told to not let baby sleep for more than 4 hours without eating for the first couple of weeks.” Other parents noted the lack of sufficient information from their care provider; one participant was recommended to purchase a book about infant sleep, while others mentioned researching on their own regarding infant wake windows.

None of the examined sociodemographic variable were associated with participants’ likelihood of receiving movement behaviour-related education in their postnatal care (Table 3).

#### Pediatric care

Findings for pediatric care were similar to that of participants’ postnatal care, with most participants (54.8%) reporting receiving information about their child’s physical activity, and only some receiving information about their child’s sedentary behaviour (25.9%) or sleep (34.9%) in their child’s pediatric care (Table 2). Over one-third (37.2%) of parents/guardians reported no mention of any of the movement behaviours in their child’s pediatric care, while few (18.9%) reported mention of all three movement behaviours. Of those who reported receiving physical activity information in their child’s pediatric care (*n* = 299), most (59.2%) indicated that this included information on tummy time, while some (29.8%) reported their child’s pediatrician discussed gross motor milestones. However, the level of detail provided by pediatricians varied, with some providing lots of detail (e.g., “Each [pediatrician appointment, the doctor] would always ask about milestones, would have baby show strength, and make continual suggestions on how to maintain/improve”), while others kept information to a minimum (e.g., “I was asked about it but had to do the research on my own”).

As the least mentioned movement behaviour in participants’ pediatric care, it is not surprising that of those reporting receiving sedentary behaviour information (*n* = 141), one-fifth (21.3%) commented further that this included content on time spent sedentary or restrained. While more participants reported the mention of screen time in their child’s pediatric care (37.6%), some participants received information conflicting with current recommendations for the early years. For example, one participant reported receiving information that “Some screen time is okay, engage together, under 2 hours (but more recently, no limits).”

For participants receiving information about their child’s sleep in their pediatric care (*n* = 190), discussion of 24-hour sleep recommendations (32.1% of parents) was more common than discussion of safe sleep recommendations (13.7% of parents). Further, some participants mentioned receiving sufficient information, including “lots of conversation on how to set them up for success for independent sleep” and that their pediatrician “would always ask how often and when baby was sleeping and give suggestions on how to have baby stretch sleep if applicable.” Others were directed to outside sources; for example, one participant mentioned “[their] doctor recommended googling wake windows for help with this topic.”

Participants’ pediatric care demonstrated the greatest differences in movement behaviour-related education as it related to sociodemographic variables. For example, compared to those identifying as white, participants who identified as an ethnic minority demonstrated greater odds of receiving sleep-related education in their pediatric care (OR = 1.649 95%CI = 1.087, 2.502, *p* = .019; Table 3). There were also significant differences in the likelihood of receiving sedentary behaviour (χ^2^ = 17.707, *p* = .013) and no movement behaviour-related education (χ^2^ = 19.788, *p* = .006) between provinces and territories. Parents from Québec were significantly more likely to receive sedentary behaviour-related education compared to parents from British Columbia (OR = 3.534, 95%CI = 1.610, 7.752, *p* = .002), Alberta (OR = 2.309, [95%CI = 1.030, 5.181, *p* = .042), Saskatchewan (OR = 2.786, 95CI = 1.098, 7.042; *p* = .031), Ontario (OR = 2.717, 95%CI = 1.416, 5.208, *p* = .003), and the Maritimes (OR = 6.757, 95%CI = 2.288, 20.000, *p* < .001). Parents in Manitoba were also significantly more likely to receive sedentary behaviour-related education compared to parents from the Maritimes (OR = 3.348, 95%CI = 1.028, 10.904, *p* = .045). Parents from British Columbia (OR = 3.458, 95%CI = 1.463, 8.175, *p* = .005), Alberta (OR = 2.903, 95%CI = 1.163, 7.244, *p* = .022), Saskatchewan (OR = 4.117, 95%CI = 1.548, 10.947, *p* = .015), Manitoba (OR = 3.422, 95%CI = 1.271, 9.212, *p* = .026), Ontario (OR = 2.469, 95%CI = 1.117, 5.460, *p* = .026), the Maritimes (OR = 7.333, 95%CI = 2.819, 19.079, *p* < .001), and the Territories (OR = 4.889, 95%CI = 1.027, 23.275, *p* = .046) were more likely to receive no movement behaviour-related education compared to parents from Québec. Finally, parents from the Maritimes were also significantly less likely to receive movement behaviour-related education compared to parents from Alberta (OR = 0.369, 95%CI = 0.169, 0.926, *p* = .033), and Ontario (OR = 0.337, 95%CI = 0.164, 0.690, *p* = .003) (see Appendix A for all ORs).

#### Sources of information about movement behaviours

When asked about where they go to get information about their child’s movement behaviours, only 228 parents/guardians (41.7%) reported reaching out to their child’s pediatrician or doctor. Most parents/guardians relied on social media (70.9%), internet websites and news articles (68.7%), and family/friends (67.6%). Few noted consulting research studies (31.3%) and mobile applications (27.2%).

### Educational needs

Participants were presented with a variety of movement behaviour topics and were asked which ones they would like additional information and/or resources about. The most sought-after physical activity-related topics included muscle- and bone-strengthening activities (63.9%), facilitating indoor active play (61.7%), and energetic play (59.3%; Table 4). Parents/guardians were also interested in sedentary behaviour-related topics such as incorporating movement into traditionally sedentary activities (68.8%) and activity ideas to break up sitting time (65.0%). Topics related to sleep in early childhood were less sought-after, with the most popular being independent sleep (41.5%) and wake windows (32.3%). Many parents/guardians were also interested in learning more about outdoor play-related topics such as how to support their child’s outdoor risky play (57.6%), outdoor activity examples in all types of weather (55.1%), and how to design an outdoor play area to support their child’s physical activity (52.1%). Participants were also able to list additional topics they wished to learn more about; most suggestions were related to sleep and included dealing with sleep regressions, transitioning to a bed, quiet time, and sleep with special needs children. Other suggestions included how to minimize screen time for young children when around older children, activities for prolonged sitting in a car, how to get outdoor play when you live in a housing type without direct outdoor access, and role modelling healthy movement behaviours (by all parents).

**Table 4:** Topics Parents Would Like to Receive More Information and Resources About

Topic	N	%
Gross motor development	178	33.3
Fine motor development	229	42.8
Fundamental movement skills	257	48.0
Tummy time	88	16.4
Energetic play	317	59.3
Muscle- and bone-strengthening activities	342	63.9
Structured physical activity ideas	293	54.8
Unstructured active play	284	53.1
Facilitating indoor active play	330	61.7
Prolonged sitting time	151	28.2
Prolonged time spent restrained in a highchair/car seat/stroller	157	29.3
Age-appropriate screen time recommendations	185	34.6
Activity ideas to break up sitting time	348	65.0
Incorporating movement into traditionally sedentary activities	368	68.8
Age-appropriate sleep recommendations	169	31.6
Wake windows	173	32.3
Safe sleep recommendations	77	14.4
Co-sleeping	157	29.3
Independent sleep	222	41.5
Sleepy cues	152	28.4
Health benefits of outdoor play	73	13.6
How to promote outdoor free play at home	234	43.7
How to dress my child(ren) for outdoor play in all weather	156	29.1
Outdoor activity examples in all types of weather	295	55.1
How to support my child(ren)’s outdoor risky play	308	57.6
How to design an outdoor play area to support my child(ren)’s physical activity	279	52.1

Note. Frequencies out of 535 respondents

When asked about the format that they would prefer to receive information about young children’s movement behaviours, the most preferred modality was social media (63.2%), followed by an online resource package (47.8%), email (46.6%), and a website with a login (43.2%). The least preferred formats included a mobile application (35.1%), a paper resource package (25.6%), and text messages (8.1%).

## Discussion

The ME & YOU needs assessment study aimed to glean greater insight regarding the education that parents and guardians living in Canada received during their prenatal, postnatal, and pediatric care, and their perceptions about their educational needs relating to movement behaviours in early childhood. While many parents reported receiving physical activity-related education in their prenatal, postnatal, and pediatric care, between 25% and 50% of participants reportedly received no movement behaviour education across these care types. Participants noted that they felt the need to research additional information from other sources and communicated their desire to learn more about movement behaviours in early childhood. These results highlight areas of improvement to better support parents/guardians in raising healthy active children from infancy and are discussed below.

This study’s finding that parents/guardians living in Canada receive limited to no information and education relating to movement behaviours in early childhood during their prenatal, postnatal, and pediatric care is concerning. In general, participants reported that they received the least movement behaviour education in their prenatal care, which is likely explained by the primary focus of this care being on the birthing parent and the health of their baby in the womb. However, this is perhaps an important time to provide parents/guardians with this information so that they feel sufficiently prepared and confident to establish healthy movement behaviour habits with their child from birth. For example, Barimani et al. [19] conducted interviews with parents (*n* = 60) in Sweden to gather parents’ experiences of their transition to parenthood; they found that lack of professional support and information inhibited a healthy transition to parenthood, while resource availability and participating in a parent education group facilitated a healthy transition. Given how overwhelmed many parents feel in the weeks, months, and years after welcoming a child [18], providing new parents with information and resources on movement behaviours in early childhood during the prenatal period may be especially important.

Parents/guardians in this study highlighted that physical activity was often discussed in their postnatal care and their child’s pediatric care, with a particular focus on tummy time and gross motor development. While these are important topics to cover during these care appointments, parents/guardians noted a prominent lack of information pertaining to their child’s sedentary behaviour and sleep. Information on these movement behaviours is important for parents/guardians to know from birth to help establish healthy development and habits that will track into later childhood and adolescence [19]. For example, if parents/guardians are unaware of the risks of excessive screen time in early childhood (e.g., increased adiposity, decreased cognitive development) [20], they may unknowingly place their child on a poorer health trajectory. Moreover, as parents/guardians noted in their educational desires, they would benefit from learning information pertaining to breaking up sedentary behaviour, independent sleep, and how to deal with sleep regressions. The necessity of including sedentary behaviour-related education for new parents/guardians is confirmed by a qualitative study by Dinkel and colleagues [21], which demonstrated that only 16.7% of parents (*n* = 12) displayed concern regarding infant restrictive devices, compared to 76.9% of healthcare providers (*n* = 12). Further, a systematic review by McDowall and colleagues [22] demonstrated that parents who were more knowledgeable about child sleep were more likely to report that their child exhibited healthy sleep behaviours. Receiving well-rounded education and information on all movement behaviours in early childhood is important, particularly from credible sources such as healthcare professionals.

While it may be encouraged for parents/guardians to seek guidance regarding their child’s movement behaviours from credible sources, such as healthcare professionals and research studies, few participants in this study reported seeking information from these sources. In the age of technology, it is not surprising that instead, many parents/guardians reported going to social media or internet articles from searching “Dr. Google”. According to a qualitative study by Moon et al., [23] mothers (*n* = 28) appreciated the ability to gather immediate and unlimited information online, including multiple opinions, quickly and anonymously. Despite the existence of credible social media accounts and online news articles, many parents/guardians may not have the critical appraisal skills needed to sort through the overwhelming amount of information (and misinformation) in the media to find proper guidance. Furthermore, given the unique development and behavioural needs of each child, online sources might not provide appropriate guidance for young children’s specific stage of development. Healthcare professionals such as obstetricians, midwives, pediatricians, and doctors should work to integrate more education on movement behaviours in early childhood into their patients’ care appointments, including providing parents/guardians with credible resources and supports (including those located online) that they can consult when questions arise.

The findings from this study surrounding the influence of province on participants’ movement behaviour-related education received in their pediatric care were expected, although it is interesting that such differences were not also observed for participants’ prenatal and postnatal care. Given healthcare is governed provincially/territorially in Canada, it is generally assumed that inter-provincial/territorial differences in care exist. These results suggest that a more coordinated effort, such as advocacy by national groups such as the Canadian Pediatric Society and the College of Family Physicians of Canada, is required to ensure that all Canadians benefit from sufficient education in their prenatal, postnatal, and pediatric care to support their child’s health.

### Research implications and future directions

This study highlights important areas for future research, including gathering perspectives of primary care providers (e.g., obstetricians, midwives, pediatricians, doctors) pertaining to their practice and whether they provide their patients with movement behaviour education for early childhood, as well as the amount of detail and external resources they offer. Gaining this insight will help confirm if, in fact, there is a widespread lack of information provided to parents/guardians as was reported in this study, or if there is simply a disconnect between what primary care providers cover and what parents/guardians retain. It would also be of value to conduct this study in other countries to see if similar results are found, or if there are countries with high success in relaying movement behaviour education to parents/guardians of young children that can act as best practice models of care. Longitudinal research studies might also help glean greater insight into how the level of movement behaviour education received during prenatal, postnatal, and pediatric care translates into parenting behaviours. Further, exploring the influence of timing of movement behaviour education delivery (e.g., during different child developmental stages) would help researchers understand when parents might be most receptive to this type of information, which could inform targeted interventions. As evidenced in this study, many parents and guardians rely on digital technology to source their movement behaviour information, via mobile applications or internet articles; as such, researchers can look to leverage digital platforms when designing educational interventions with parents of young children.

This research also has implications for primary care. Specifically, care providers across Canada should begin integrating information about movement behaviours into their patients’ prenatal, postnatal, and pediatric care to increase their awareness of the importance of establishing healthy movement behaviours from birth, and to help parents feel more confident in supporting their child to meet the *Canadian 24-Hour Movement Guidelines for the Early Years*. This practice would help ensure that parents/guardians are receiving this health information from a credible source, as well as create opportunities for parents/guardians to ask questions. Other countries that have successfully implemented, and transitioned multi-component interventions into standard practice, such as the Melbourne Infant Feeding and Nutrition Trial in Australia [24], can offer guidance on how research and primary practice can work together to achieve common health goals [25]. This program was an early childhood obesity prevention intervention, delivered via Maternal and Child Health Centres to first-time parents, and focused on parenting skills that supported the development of positive diet and physical activity behaviours, and reduced sedentary behaviours in infants from 3 to 18 months of age. The program has been running for over 15 years and serves as a sustainable model of care for first-time parents.

### Strengths and limitations

While this study has several strengths, including its sample size with demographic representation across provinces/territories and ethnicities, there are some limitations to consider. First, as items in this survey were self-reported, some questions (e.g., those relating to participants’ prenatal, postnatal, and pediatric care) are subject to recall bias and may not be a true representation of the actual care received. This might have been especially true for parents/guardians whose youngest child was 4 years old, or for participants who were not the birthing parent. Second, the proportion of participants elaborating on their movement behaviour education received in their prenatal, postnatal, or pediatric care via text responses to provide greater contextual information on topics covered was limited to those who chose to do so; as such, it is likely that these proportions are an underrepresentation of the actual percentage of topics covered. Finally, participants were primarily female, highly educated, from double parent households, and had middle-to-upper class annual household incomes; as such, generalizability to other populations, including lower socioeconomic status and single parent groups is limited.

## Conclusion

This study provides the first national evidence of the educational background and needs pertaining to movement behaviours in early childhood among parents/guardians living in Canada. Findings highlighted gaps and inconsistencies in education relating to movement behaviours in early childhood in participants’ prenatal, postnatal, and pediatric care, with sedentary behaviour and sleep education often overlooked. Parents/guardians expressed their desire to learn more about a variety of movement behaviour topics and were most curious to better understand how to minimize young children’s sedentary behaviour. In light of this evidence, there is a great opportunity for integration of movement behaviour education across care types. Supporting the integration of evidence-based and consistent guidance on young children’s movement behaviours into prenatal, postnatal, and pediatric care will help ensure parents/guardians are well equipped to foster their child’s development of healthy movement habits.

### Electronic supplementary material

Below is the link to the electronic supplementary material.

Supplementary Material 1

## Data Availability

No datasets were generated or analysed during the current study.

## Abbreviations

CI: Confidence interval
*M*: Mean
ME & YOU: Movement Education for parents of YOUng children
MVPA: Moderate-to-vigorous physical activity
OR: Odds ratio
*SD*: Standard deviation
