# Supplementary material for: Movement behaviour education for parents in prenatal, postnatal, and pediatric care in Canada: A needs assessment
Source: BMC Pediatr. 2024 Mar 8;24:164. doi: 10.1186/s12887-024-04630-4 (PMC10921676; doi:10.1186/s12887-024-04630-4)
Supplement: Supplementary file 1 — Supplementary Material 1 [file 12887_2024_4630_MOESM1_ESM.docx]

| Appendix A. *Influence of Sociodemographic Characteristics on Participants’ Movement Behaviour-Related Education Received in their Prenatal, Postnatal, and Pediatric Care.* | | | | |
| --- | --- | --- | --- | --- |
| **Prenatal Care** | | | | |
| **Sociodemographic Characteristic** | **Physical Activity** | **Sedentary Behaviour** | **Sleep** | **None** |
| Ethnicity  White (ref)  Ethnic minority | 1.272 (0.834, 1.942), .264 | 1.247 (0.793, 1.960), .339 | 1.325 (0.857, 2.048), .205 | 0.725 (0.475, 1.107), .137 |
| Income  Less than $40,000  $40,000 - $79,999  $80,000 - $119,999  $120,000+ (ref) | 0.597 (0.221, 1.611), .309  0.527 (0.211, 1.318), .171  0.458 (0.191, 1.096), .079 | 0.706 (0.261, 1.910), .493  0.460 (0.182, 1.162), .100  0.531 (0.223, 1.263), .152 | 1.103 (0.398, 3.060), .851  1.056 (0.412, 2.708), .909  0.900 (0.367, 2.206), .819 | 1.741 (0.634, 4.783), .282  1.842 (0.723, 4.692), .201  2.185 (0.896, 5.330), .086 |
| Education  High School  College  University  Graduate school (ref) | 1.308 (0.423, 4.042), .641  **2.824 (1.525, 5.229),<.001**  1.064 (0.718, 1.576), .758 | 1.262 (0.372, 4.820), .709  **2.065 (1.115, 3.827), .021**  1.186 (0.770, 1.827), .438 | 0.400 (0.086, 1.861), .243  1.600 (0.871, 2.939), .130  1.199 (0.795, 1.806), .387 | 0.667 (0.216, 2.058), .481  **0.389 (0.209, 0.723), .003**  0.881 (0.598, 1.299), .523 |
| Province  Ontario (ref)  Alberta  British Columbia  Manitoba  Quebec  Saskatoon  Maritimes  Territories | 1.094 (0.577, 2.075), .783  0.871 (0.489, 1.551), .638  0.862 (0.404, 1.837), .700  1.231 (0.633, 2.391), .540  1.308 (0.613, 2.788), .487  0.533 (0.257, 1.105), .091  1.846 (0.299, 11.381), .509 | 1.384 (0.702, 2.727), .348  0.878 (0.460, 1.676), .693  1.057 (0.465, 2.403), .895  1.951 (0.983, 3.874), .056  1.649 (0.751, 3.619), .213  0.580 (0.248, 1.355), .208  1.691 (0.273, 10.943), .573 | 1.507 (0.780, 2.910), .111  0.922 (0.469, 1.714), .601  0.897 (0.396, 2.029), .603  1.384 (0.696, 2.752), .177  1.793 (0.831, 3.871), .069  0.933 (0.445, 1.952), .573  3.228 (0.521, 19.985), .104 | 0.830 (0.438, 1.572), .567  1.108 (0.626, 1.964), .725  1.050 (0.497, 2.219), .898  0.657 (0.336, 1.285), .220  0.878 (0.412, 1.872), .737  1.575 (0.783, 3.168), .203  0.622 (0.101, 3.835), .609 |
| **Postnatal Care** | | | | |
| **Sociodemographic Characteristic** | **Physical Activity** | **Sedentary Behaviour** | **Sleep** | **None** |
| Ethnicity  White (ref)  Ethnic minority | 0.948 (0.614, 1.464), .811 | 1.208 (0.778, 1.875), .401 | 1.079 (0.706, 1.649), .726 | 0.983 (0.623, 1.552), .943 |
| Income  Less than $40,000  $40,000 - $79,999  $80,000 - $119,999  $120,000+ (ref) | 1.246 (0.490, 3.166), .645  0.651 (0.358, 1.182), .159  0.955 (0.602, 1.514), .844 | 0.890 (0.350, 2.266), .808  0.825 (0.435, 1.567), .557  1.161 (0.731, 1.845), .526 | 0.755 (0.312, 1.830), .534  0.903 (0.498, 1.636), .903  0.927 (0.593, 1.451), .927 | 1.306 (0.526, 3.242), .565  1.500 (0.813, 2.766), .194  0.926 (0.567, 1.512), .758 |
| Education  High School  College  University  Graduate school (ref) | 0.537 (0.179, 1.609), .266  1.252 (0.673, 2.332), .478  1.320 (0.773, 1.972), .176 | 1.267 (0.407, 3.949), .683  1.639 (0.882, 3.046), .118  1.313 (0.866, 1.989), .199 | 0.365 (0.099, 1.351), .131  1.728 (0.942, 3.196), .077  1.350 (0.912, 1.999), .134 | 1.791 (0.603, 5.324), .294  0.871 (0.461, 1.649), .672  **0.645 (0.422, 0.986), .043** |
| Province  Ontario (ref)  Alberta  British Columbia  Manitoba  Quebec  Saskatchewan  Maritimes  Territories | 1.805 (0.919, 3.546), .087  1.650 (0.908, 2.998), .100  1.333 (0.620, 2.863), .462  0.928 (0.439, 1.961), .845  1.898 (0.843, 4.269), .122  1.129 (0.575, 2.218), .725  3.300 (0.360, 30.245), .291 | 1.448 (0.746, 2.810), .274  1.533 (0.845, 2.780), .160  1.224 (0.558, 2.687), .614  1.389 (0.640, 3.018), .406  1.870 (0.866, 4.040), .111  0.726 (0.338, 1.561), .412  1.496 (0.242, 9.266), .665 | 1.604 (0.844, 3.046), .149  1.615 (0.907, 2.874), .103  0.922 (0.432, 1.967), .834  0.719 (0.331, 1.561), .404  2.027 (0.937, 4.383), .073  0.800 (0.403, 1.588), .523  1.976 (0.321, 12.184), .463 | 0.637 (0.311, 1.304), .217  0.657 (0.348, 1.242), .196  0.891 (0.402, 1.973), .776  1.471 (0.689, 3.137), .318  0.596 (0.251, 1.417), .242  1.028 (0.511, 2.067), .939  0.466 (0.051, 4.277), .499 |
| **Pediatric Care** | | | | |
| **Sociodemographic Characteristic** | **Physical Activity** | **Sedentary Behaviour** | **Sleep** | **None** |
| Ethnicity  White (ref)  Ethnic minority | 1.050 (0.694, 1.588), .816 | 1.159 (0.736, 1.824), .525 | **1.649 (1.087, 2.502), .019** | 0.751 (0.487, 1.157), .194 |
| Income  Less than $40,000  $40,000 - $79,999  $80,000 - $119,999  $120,000+ (ref) | 1.250 (0.533, 2.933), .608  0.866 (0.488, 1.534), .621  0.740 (0.478, 1.147), .178 | 2.265 (0.981, 5.227), .055  1.005 (0.525, 1.925), .987  1.039 (0.634, 1.704), .880 | 2.340 (1.021, 5.362), .044  0.896 (0.490, 1.638), .721  0.753 (0.370, 1.208) .753 | 0.535 (0.207, 1.385), .197  1.283 (0.721, 2.284), .397  1.264 (0.811, 1.969), .300 |
| Education  High School  College  University  Graduate school (ref) | 0.597 (0.200, 1.787), .357  1.041 (0.581, 1.867), .892  1.086 (0.740, 1.594), .674 | 1.951 (0.621, 6.128), .252  1.277 (0.657, 2.483), .471  **1.561 (1.007, 2.419), .046** | 1.241 (0.399, 3.859), .709  1.595 (0.878, 2.897), .125  1.371 (0.916, 2.051), .125 | 1.983 (0.662, 5.937), .221  0.801 (0.438, 1.464), .470  0.833 (0.563, 1.233), .360 |
| Province  Ontario (ref)  Alberta  British Columbia  Manitoba  Quebec  Saskatchewan  Maritimes  Territories | 0.821 (0.432, 1.559), .546  0.756 (0.427, 1.340), .338  0.758 (0.359, 1.604), .469  1.878 (0.939, 3.755), .075  0.714 (0.341, 1.495), .371  **0.449 (0.228, 0.887), .021**  0.449 (0.073, 2.771), .389 | 1.175 (0.581, 2.377), .653  0.769 (0.391, 1.515), .448  1.349 (0.602, 3.020), .467  **2.716 (1.417, 5.206), .003**  0.976 (0.421, 2.265), .956  0.434 (0.171, 1.104), .080  1.880 (0.303, 11.676), .498 | 1.135 (0.590, 2.183), .704  0.861 (0.472, 1.569), .625  0.633 (0.276, 1.455), .282  1.827 (0.969, 3.443), .062  0.918 (0.423, 1.990), .828  0.503 (0.231, 1.094), .083  1.173 (.0190, 7.239), .864 | 1.176 (0.607, 2.278), .632  1.400 (0.781, 2.513), .259  1.386 (0.646, 2.972), .402  **0.405 (0.183, 0.896), .026**  1.667 (0.790, 3.519), .180  **2.970 (1.495, 5.901), .002**  1.320 (0.214, 8.157), 765 |
